# Effects of online brain training on self‐reported mental health symptoms for generally healthy adults during the Covid‐19 pandemic

**DOI:** 10.1002/brb3.2853

**Published:** 2022-12-21

**Authors:** Sarah A. Laane, Lori G. Cook, Jeffrey S. Spence, Michelle N. Harris, Sandra Bond Chapman

**Affiliations:** ^1^ Center for BrainHealth, Department of Behavioral and Brain Sciences The University of Texas at Dallas Dallas Texas USA; ^2^ Program of Criminology and Criminal Justice, School of Economic, Political and Policy Sciences The University of Texas at Dallas Richardson Texas USA

**Keywords:** anxiety, cognition, depression, digital health, mental health, pandemic, prevention, public health, stress

## Abstract

**Background:**

The cognitive training Strategic Memory Advanced Reasoning Training (SMART) has been shown to improve symptoms of depression, anxiety, and stress when completed using in‐person delivery, but mental health outcomes have not yet been studied for online delivery of SMART.

**Methods:**

Data was analyzed from 145 generally healthy adults participating in the BrainHealth Project pilot study who had access to 12 weeks of online self‐paced SMART and self‐reported mental health symptoms on the Depression Anxiety Stress Scale (DASS‐21) pre‐ and post‐training. We utilized linear models to examine the change in self‐reported symptoms of depression, anxiety, and stress following the 12‐week training period and to explore the influence of age, gender, and education on changes in symptomatology. Data from 44 participants who completed a follow‐up DASS‐21 6 months after completing SMART was used to explore the lasting impact of the training.

**Results:**

Improvements in depression, anxiety, and stress symptoms were observed following online SMART, evidenced by a significant decrease in self‐reported symptoms on the DASS‐21. Improvement in self‐reported mental health symptomatology was maintained or continued to improve 6‐month post‐training. No significant effect of gender was observed, but findings motivate additional exploration of the effects of education and age.

**Conclusion:**

Online SMART should be considered a low‐cost, high‐impact approach for supporting public mental health for generally healthy adults.

## Graphical abstract

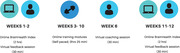

## 1 BACKGROUND

In 2019, nearly one in five adults in the United States reported living with a mental illness (Substance Abuse and Mental Health Services Administration, 2020). Of this group, 26% reported a perceived unmet need for mental health services, which is a higher percentage than has been reported in the past 10 years of this survey. This crisis has likely worsened during the Covid‐19 pandemic. Recent studies of the general population indicate lower levels of reported psychological well‐being and increased depressive and anxiety symptomatology (Vindegaard & Benros, 2020). Those with preexisting mental illnesses also reported an increase in symptomatology. Additionally, patients who contracted Covid‐19 showed high levels of post‐traumatic stress and depressive symptoms (Vindegaard & Benros, 2020). An examination of 911 calls found an increase in mental health–related calls to police during the pandemic compared to the pre‐pandemic call ratio (Konkel et al., 2022). More resources are needed to help alleviate this growing mental health crisis at the earliest point possible. One way scientists can help is to identify novel low‐cost, high‐impact approaches to intercept those beginning to experience a decline in mental health before reaching a point of disorder requiring treatment from an already overloaded system.

Emerging evidence suggests that interventions that target cognitive processes, such as reasoning, attention, and problem‐solving, may also provide mental health benefits. A notable example is Strategic Memory Advanced Reasoning Training (SMART), which is a cognitive training program that targets higher order cognitive control by targeting functions mediated by the frontal networks of the brain (Chapman et al., 2021; Gamino et al., 2009a; Vas et al., 2016). Specifically, SMART trains participants to use tactical strategies in the areas of strategic attention (ex. identifying important vs. unimportant information and filtering out distracting or unimportant information), integrated reasoning (ex. synthesizing big‐picture ideas from important detail‐level information), and innovation (ex. generating multiple new ideas, solutions, and perspectives). SMART has been shown to promote improvements in both trained and untrained areas of cognitive functioning, including strategic attention (Gamino et al., 2009b, 2009a), innovation (Chapman et al., 2017; Young et al., 2021), meaning abstraction (Anand et al., 2010), gist‐reasoning/integrated reasoning (Gamino et al., 2010; Vas et al., 2011; Young et al., 2021), fact‐learning (Gamino et al., 2010), working memory (Cook et al., 2014, 2020; Gamino et al., 2009b; Vas et al., 2011), initiation (Gamino et al., 2009b), cognitive switching (Anand et al., 2010; Cook et al., 2020), and real‐life executive function behaviors (Cook et al., 2020; Gamino et al., 2009b). Beyond yielding improvements in areas of cognitive function, as it was designed to address, SMART has also demonstrated unexpected benefits in mental health symptomatology (Vas et al., 2016; Young et al., 2021).

More specifically, Vas et al. (2016) examined the effects of SMART compared to a control program for 60 veterans and civilians with a history of TBI. The experimental group received 18 h of in‐person small group SMART, whereas the control group received brain health education via a program called Brain Health Workshop (BHW). BHW included science‐backed education on brain anatomy and injury, cognitive function, and brain healthy habits. The SMART group showed significant gains in cognitive performance over the BHW group in the areas of gist reasoning, switching (executive function), and memory (recall and delayed recall). Additionally, and of interest to this investigation, the SMART group also had a significant reduction in symptoms of depression, as measured by the Beck Depression Inventory 2nd edition, and stress‐related symptoms, as measured by the PTSD Checklist, compared to the BHW group. In this way, Vas and colleagues demonstrated that cognitive training could yield mental health benefits for those with persistent effects of TBI.

Positive effects on mental health following SMART have also been observed in nonclinical populations. Young et al. (2021) examined in‐person SMART across three study groups: two groups of military personnel and veterans and one group of healthy police officers. The military personnel and veteran groups (*N* = 425) received 6–10 h of SMART across a 2‐day training, whereas the police officer group (*N* = 75) completed 9 h of SMART across 3 days of training. Similar to the previous findings, all groups showed improvements in cognitive performance following SMART. The military personnel and veteran groups showed significant gains in integrated reasoning and innovation, and the police officer group showed significant gains in strategic attention and innovation. Additionally, all three groups showed improvements in mental health symptomatology. The first military/veteran group had a significant decrease in stress, depression, and anxiety symptoms following SMART, as measured by the Depression Anxiety Stress Scale‐21 (DASS‐21). This group had a 3–4 h strategy review and practice session 1‐month post‐training and repeated this assessment 1‐month post‐training (with 69 respondents) and 4‐months post‐training (with 77 respondents). Results for the follow‐up assessment showed significant improvements were sustained or continued to improve up to 4‐months post‐training. The second military/veteran group had a significant decrease in depression, as measured by the Beck Depression Index, and in anxiety, as measured by the Beck Anxiety Index. The police group had a significant reduction in symptoms of depression and stress, but not anxiety, reported on the DASS‐21.

This body of evidence suggests that SMART training not only improves areas of cognitive performance but also generalizes to mental health benefits. However, SMART's effects on mental health outcomes have not yet been measured using an online/internet‐based delivery. An online delivery model would be an ideal low‐cost model of distribution and could improve access for those with limited time or resources to travel to providers. There is evidence that online cognitive training that targets basic cognitive processes, such as processing speed, working memory, and attentional control, may provide benefits for mental health symptomatology (Calkins et al., 2014; Ronghua et al., 2015; Wolinsky et al., 2009); however, this has not yet been explored for a training that targets higher order cognitive controls such as SMART.

Mental health professionals are already using interventions that incorporate cognitive processes, such as cognitive behavioral therapy (CBT) and cognitive processing therapy, as a treatment method for those with mental illness (American Psychological Association, 2017; Fenn & Byrne, 2013). However, these interventions require direct implementation from a licensed professional and are generally reserved for those who have been diagnosed with a mental illness rather than used as a preventative measure. These types of interventions explore the connection between thoughts, emotions, and behaviors and therefore engage cognition through helping clients identify and change their beliefs, understanding, and/or conceptualization of specific psychological experiences (ex. working through trauma, addiction, obsessive/intrusive thoughts, etc.). Cognitive training like SMART differs greatly from these approaches, as strategies are not designed to help patients process specific psychological experiences. Rather, SMART provides training on strategies that engage frontal‐lobe‐mediated cognitive processes, such as strategic attention, gist‐reasoning (big‐picture thinking), and problem‐solving. The individual participant is then responsible for how they chose to apply these practical strategies in their daily lives. In this way, SMART is ideal for scaling to the population level, as the training modules themselves are not individuated for specific needs.

An online SMART program that generalizes to provide psychological benefits could be a tool utilized to meet rising mental health concerns among the public. Study aims were as follows:

Aim 1: To examine the benefit of online tactical brain strategy training (i.e., SMART) on reported symptoms of depression, anxiety, and stress in a generally healthy adult population during the Covid‐19 pandemic.

Aim 2: To explore the influence of age, gender, and education on changes in depression, anxiety, and stress following the completion of online SMART.

Aim 3: To identify if changes in depression, anxiety, and stress are maintained 6‐months post‐training with continued access to SMART resources and practice.

We hypothesized that depressive, anxiety, and stress symptoms would decrease upon the completion of the online cognitive training SMART. Additionally, we hypothesized that there would not be a significant effect of age, gender, or education on the decrease of depression, anxiety, and stress symptoms following the completion of online SMART. Finally, we hypothesized that improvements from baseline symptoms of depression, anxiety, and stress would be maintained 6‐months post‐training.

## 2 METHODS

Participant data for this article came from the BrainHealth Project (Chapman et al., 2021). The BrainHealth Project (i.e., the project) is a longitudinal study launched by the Center for BrainHealth designed to define, measure, and improve brain health over the lifespan. Participants in the project have the opportunity to engage with different types of training over the course of their time in the study; however, all participants begin by engaging with online SMART. This provides an ideal opportunity to explore the current aims. Specifically, the data used for this article is from the 179 project pilot participants. Importantly, this data was collected in early to mid‐2020, during the onset of the Covid‐19 pandemic.

### 2.1 Protocol

BrainHealth Project pilot participants were recruited via word of mouth and email advertising. Inclusion criteria included English proficiency, ability to access the internet, and being at least 18‐years old. Exclusion criteria included those with brain injury or neurological disorder, psychotic disorders or uncontrolled psychiatric disorders, and/or uncontrolled health issues.

**FIGURE 1:**
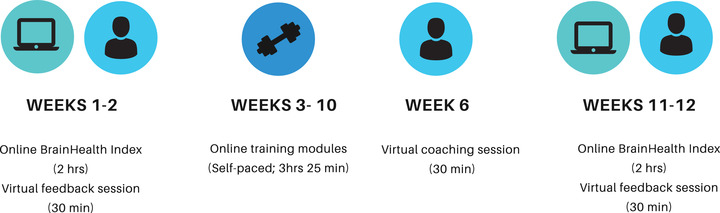
Recommended BrainHealth Project pilot training cadence

Participants completed assessment, SMART training, and coaching over a 3‐month period using a personalized dashboard on the online BrainHealth Project platform. Although the online trainings were self‐paced, participants were given a recommended 12‐week study cadence to follow (Figure 1). The first 2 weeks were allotted for time to complete a baseline assessment called the BrainHealth Index (BHI) and to receive feedback on initial assessment performance through a 30‐min video call with a brain health coach. The BHI is a comprehensive measure of brain health that includes measures in the areas of cognitive performance, social interaction, well‐being and mood, and daily‐life activities. This study focuses specifically on the BHI's mood measure, the DASS‐21 (Lovibond & Lovibond, 1995). The DASS‐21 has subscales that measure self‐reported symptoms of depression, anxiety, and stress, as well as an overall dimension of psychological distress (Henry & Crawford, 2005; Lovibond & Lovibond, 1995). The DASS‐21 asks seven questions for each subscale. Participants answer questions on a Likert scale of Never—0, Sometimes—1, Often—2, and Almost Always—3. The depression subscale asks questions relating to feelings of self‐despair, gloom, and lack of initiation. For example, “Over the past week, I couldn't seem to experience any positive feeling at all” and “Over the past week, I found it difficult to work up the initiative to do things.” The anxiety subscale includes questions about feeling worried or panicked as well as physical sensations, such as dryness of mouth, pounding of heart, or difficulty breathing. For example, “Over the past week, I felt I was close to panic” and “Over the past week, I was aware of the action of my heart in the absence of physical exertion (e.g., sense of heart rate increase, heart missing a beat).” The stress subscale asks questions related to feelings of over‐arousal, irritation, and tension. For example, “Over the past week, I found it hard to wind down” and “Over the past week, I tended to overreact to situations.”

Weeks 3–10 of the study were allotted for completion of the online training modules. Training modules utilized a microburst learning approach (Vaughn, Baker, & Dewitt, 1998) by including short videos in which researchers described the SMART strategies, provided information about the science behind the strategies, and provided examples of how to apply the strategies in everyday life. Training videos were interspersed with short open‐ended or multiple‐choice reflection questions to allow for participant engagement. After viewing a brief overview video to introduce the training, participants began the training modules. The first four modules (∼30 min each) introduced SMART strategies in the areas of strategic attention, integrated reasoning, and innovation and provided opportunities via the reflection questions to practice utilizing strategies. The next three modules (∼20 min each) link SMART strategies with principles of stress management and resiliency research and techniques. The final two modules provided information on sleep and sleep hygiene (∼20 min) and information on Covid‐19 (∼5 min). Approximately 6 weeks into the training period, participants were offered the opportunity to complete a 30‐min virtual coaching session with a master's level clinician to clarify SMART strategies and to help participants generate personal goals using the strategies. At weeks 11–12, participants completed a post‐training BHI assessment and feedback session. For additional information on the training protocol, see Chapman et al. (2021).

After completing this initial 12‐week training period, participants were given the opportunity to continue in the project. Participants who consented to continue in the larger scale longitudinal Project began by completing another BHI assessment 6 months after the initial post‐assessment (9 months after baseline assessment). During this training application phase, participants had continued access to the online training dashboard including the ability to redo training modules, completion of additional 15‐min weekly practice modules, access educational resources and recommended daily goals, and/or meet with a coach for an additional coaching session.

### 2.2 Participants

**FIGURE 2:**
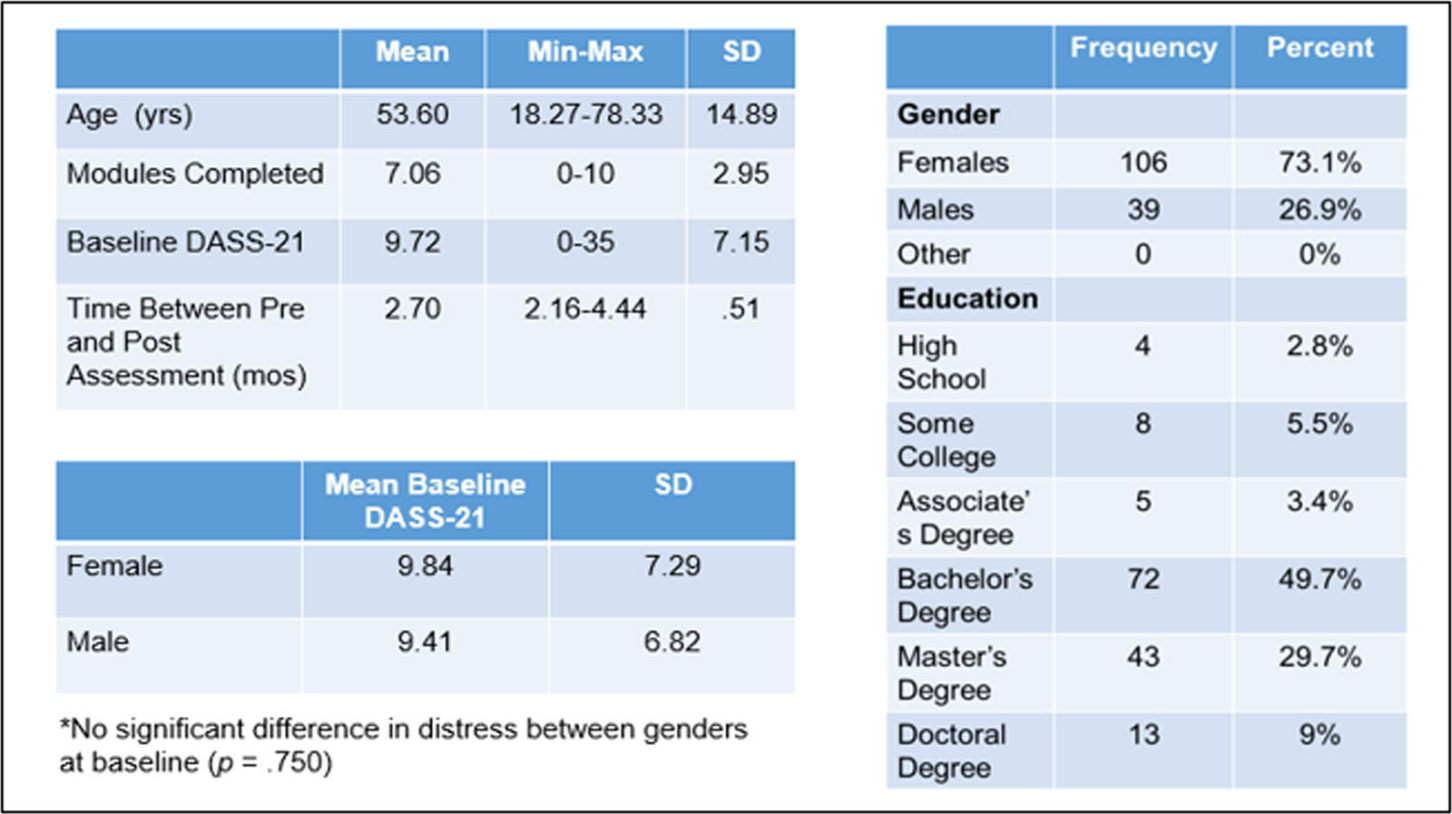
Participant demographics

Of the 179 healthy adults who participated in the Project pilot, 145 were included in the present study. Participants were excluded from this analysis if they had not completed at least two assessment time points. Participant age ranged from 18 to 78 years, with a mean of 53.60 years old (*SD* = 14.89). Participants included 106 females and 39 males and were predominantly college educated with a bachelor's degree or higher (see Figure 2). Although the sample consisted of healthy adults, participants reported some symptoms of psychological distress at baseline on the DASS‐21 (*M* = 9.72, *SD* = 7.15). There was not a statistically significant difference (*p* = .750) for baseline levels of psychological distress between female (*M* = 9.84, *SD* = 7.29) and male (*M* = 9.41, *SD* = 6.82) participants on the DASS‐21.

## 3 RESULTS

**FIGURE 3:**
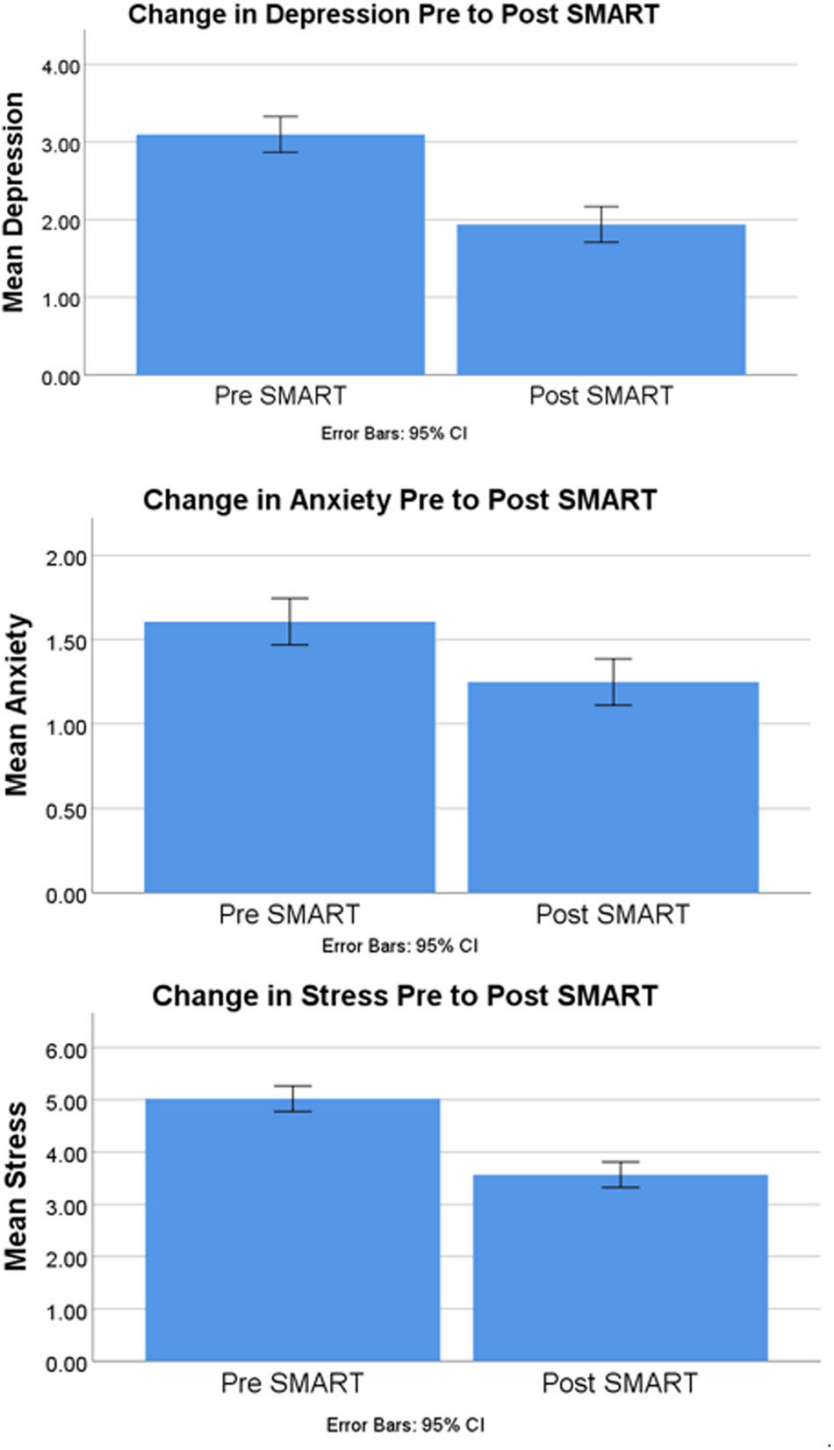
Statically significant decrease in self‐reported symptoms of depression (*p* < .001), anxiety (*p* = .011), and stress (*p* < .001)

All analyses were completed using version 26 of the software Statistical Package of the Social Sciences (SPSS), with significance levels set at .05. The interest of aim 1 was to measure mean change in depression, anxiety, and stress from pretraining to post‐training. A within‐subjects paired samples *t*‐test indicated depression scores post‐training were significantly improved *t*(144) = 4.99, *p* < .001, 95% *CI* [.70, 1.62], as manifested by a lower number of depressive symptoms endorsed following SMART (*M* = 1.94, *SD* = 2.44) as compared to baseline pretraining reporting (*M* = 3.10, *SD* = 3.06). Another paired samples *t*‐test indicated a significant improvement in anxiety symptoms following online SMART, *t*(144) = 2.57, *p* = .011, 95% *CI* [.08, .64], such that post‐training anxiety symptom means (*M* = 1.25, *SD* = 1.81) were significantly lower than pretraining anxiety symptom means (*M* = 1.61, *SD* = 1.99). A final paired samples *t*‐test indicated a significant improvement in symptoms of stress *t*(144) = 5.88, *p* < .001, 95% *CI* [.97, 1.94], such that mean stress symptoms post‐training (*M* = 3.57, *SD* = 2.82) were significantly lower than pretraining (*M* = 5.02, *SD* = 3.51). These findings are depicted in Figure 3.

A univariate general linear model was used to explore the influence of age, gender, and education on changes in depression, anxiety, and stress (Aim 2). The results are summarized in Table 1. Because of the relatively small sample size of those who reported an education level of high school (*n* = 4), some college (*n* = 8), and associate's degree (*n* = 5), these three levels of education were combined to an education level of *less than a bachelor's degree* (i.e., <Bachelor's) for this analysis. A significant effect of education (*p* = .009) was observed. A pairwise comparison was completed to explore the effect of education, which revealed that those with a bachelor's degree did not show as much improvement in depression symptoms as those with a master's degree (*p* = .002) or those with a doctoral degree *(p* = .047). See Table 2. Gender did not show a significant effect on symptoms of depression (*p* = .357), anxiety (*p* = .936), or stress (*p* = .631) following online SMART. A relationship nearing significance of age on change in stress was also observed (*p* = .053); this could indicate a trend that stress symptoms may have decreased more for those of younger ages.

**TABLE 1:** Effect of age, gender, and education on changes in self‐reported depression, anxiety, and stress symptoms

	**B**	**Std. error**	***p*‐Value**
**Depression**			
Age	−.022	.016	.165
Gender	.478	.517	.357
Education			.009^a^
**Anxiety**			
Age	.001	.010	.926
Gender	−.026	.324	.936
Education			.813
**Stress**			
Age	−.034	.017	.053^b^
Gender	.273	.567	.631
			.522

^a^ Indicates significance at p < .05.

^b^ Indicates nearing significance at p < .05.

**TABLE 2:** Effect of education level on changes in self‐reported depression symptoms

	**Mean difference**	**Std. error**	***p*‐Value**
**<Bachelor's**			
Bachelor's	.870	.744	.244
Master's	−.831	.790	.294
Doctoral	−.780	1.017	.444
**Bachelor's**			
<Bachelor's	−.870	.744	.244
Master's	−1.702	.527	.002^a^
Doctoral	−1.650	.825	.047^a^
**Master's**			
<Bachelor's	.831	.790	.294
Bachelor's	1.702	.527	.002^a^
Doctoral	.051	.867	.953
**Doctoral**			
<Bachelor's	.780	1.017	.444
Bachelor's	1.650	.825	.047^a^
Master's	−.051	.867	.953

^a^ The mean difference is significant at the .05 level.

Using a repeated measures ANOVA, lasting benefits in decreased depression, anxiety, and stress symptoms for participants who completed a 6‐month post‐training (9‐month post‐baseline) BHI assessment, *n* = 44 was also examined (aim 3). Results are summarized in Table 3. For this sample, reported depression (*p* = .001) and stress (*p* = .006) symptoms decreased significantly post‐training (3‐month post‐baseline). These significant decreases in depression (*p* = .008) and stress (*p* = .005) symptoms were maintained 6‐months post‐training (9‐month post‐baseline). Although mean anxiety symptoms decreased from pretraining (*M* = 1.39, *SE* = .31) to post‐training (*M* = 1.05, *SE* = .30), no significant effect was observed (*p* = .157). However, a significant decrease (*p* = .033) in self‐reported anxiety symptoms was observed 6‐months post‐training (9‐month post‐baseline).

**TABLE 3:** Changes in mean depression, anxiety, and stress symptoms for those participants who completed a 6‐month post‐training BrainHealth Index (BHI) (*n* = 44)

	**Pre‐training (**mean [SE])	**Post‐training (**mean [SE])	**6‐month post‐training (**mean [SE])	**p‐Value (**post/6‐month post)
**Depression**	2.84 (.48)	1.36^a^ (.29)	1.52^a^ (.33)	.001/.008
**Anxiety**	1.39 (.31)	1.05 (.30)	.86^a^ (.27)	.157/.033
**Stress**	4.11 (.51)	3.00^a^ (.48)	2.73^a^ (.42)	.006/.005

^a^ Indicates mean significantly different from pretraining score at the .05 level.

## 4 DISCUSSION

Findings indicated that participants who completed online SMART experienced similar mental health benefits to those previously demonstrated after in‐person SMART programs. Specifically, participants reported significant improvement in mental health symptomatology, as evidenced by a reduction in depression, anxiety, and stress symptoms following approximately 3 months of online cognitive SMART training access. Our finding that completion of online SMART yielded measurable improvements on psychological distress provides support that higher order cognitive control trainings, such as SMART, may be a tool to help those experiencing preclinical symptoms, particularly for those experiencing depression and stress symptomatology. Cognitive‐based interventions, such as CBT, are already used to support individuals with a clinically identified mental illness by helping patients challenge core beliefs, define problems, and gain skills to manage these problems (Fenn & Byrne, 2013; Hofmann et al., 2012). SMART may help arm prodromal populations, those who perhaps report mild symptoms but are below the threshold for clinically significant mental health diagnosis, with similar problem‐solving strategies that could be beneficial in intercepting those beginning to struggle with their mental health before developing an illness/disorder. For example, SMART's strategic attention strategies encourage participants to set aside focused time for goal‐directed behaviors, whereas integrated reasoning and innovation strategies provide tools to enhance problem‐solving such as possibility thinking (e.g., generating multiple solutions and/or viewpoints) and big‐picture perspective taking (e.g., “Zooming out” to integrate multiple ideas into a big‐picture theme or perspective). By providing cognitive training supports such as SMART to the general population, it may be possible to relieve some of the burdens on an already overburdened mental health system, as a provision of such tools may decrease symptoms of psychological distress before an individual reaches a clinical threshold for mental illness.

Past in‐person SMART utilized seminar‐style trainings in large or small groups. For example, Vas et al. (2016) trained police officers in 9 h of SMART across 3 training days. It was possible that the increased social interaction of meeting with a similar group of people (e.g., other veterans, other police officers, and others with brain injury) contributed to positive mental health outcomes. However, the findings of this investigation support that the cognitive training itself, and not the increased socialization opportunity from in‐person meetings, enhanced mental health outcomes following the training. As opposed to other cognitive training models in which training only occurs during structured sessions (ex. clicking keyboard commands in response to stimuli, answering generic reasoning or problem‐solving questions in real‐time, etc.), SMART is designed to teach frontal lobe engaging strategies that can be utilized during everyday tasks. Therefore, the context for cognitive training practice in SMART is large during everyday tasks. During past in‐person trainings, group members could share specific examples with one another of using strategies in a context relevant to that group. For example, retired veterans might discuss strategic attention strategies in the context of reducing distracting stimuli at home (e.g., turning off TV or radio when speaking with spouse), whereas working police officers might discuss strategic attention strategies in the context of locating a less stimulating environment to complete a crime report. Although the self‐paced online SMART did not provide this same opportunity to discuss context for training with a familiar group, the online SMART curriculum compensated for this difference by (1) including modules that provided examples of using SMART strategies in contexts that are shared among most people (e.g., stress management and sleep hygiene), (2) including reflection questions within modules encouraging participants to practice strategies on real‐life goals (e.g., use the Power of Two strategy to create your daily to‐do list), and (3) providing one‐on‐one virtual coaching sessions during which coaches could guide participants in implementing strategies into their own unique routines.

In considering SMART as a possible public health tool, it is important to understand its efficacy for the population as a whole. Our examination did not find a significant effect of gender on changes in symptoms of depression, anxiety, and stress following online SMART. Therefore, SMART could be a viable tool in supporting mental health for both women and men. This is a crucial finding, as gender‐based differences have been observed in mental health presentation, including differences in mental illness risk factors, disorder prevalence, and risk factor reduction (Afifi, 2007). Men and women also often report differences in help‐seeking, including differing levels of interest and access to mental health services (Afifi, 2007; Drapeau et al., 2009; Gleasona et al., 2014). Our results indicated that although SMART is a viable option for both men and women, women enrolled in the BrainHealth Project pilot at about a 3‐to‐1 ratio as compared to men, indicating women may have more interest in supporting their brain health through this holistic lens.

Additional exploration is needed to examine the possible effects of education levels on depression following SMART. We found a significant effect of education, such that those with a bachelor's degree did not show as much improvement in depression symptoms following SMART as those with a master's or doctoral degree. However, we do not believe this finding necessarily indicates that SMART is more appropriate for those with higher education levels. To the contrary, like those with postgraduate degrees, we also did not find a significant effect of education on changes in depression for those with less than a bachelor's degree. We cautiously interpreted this finding to suggest that it is not a construct of the training (ex. vocabulary level, relevancy of examples) that drives this observation but instead could perhaps indicate preexisting traits shared by those with higher levels of education. It is also important to consider that a majority of participants in the sample held a high level of education; therefore, continued exploration with greater homogeneity of education levels in the sample is warranted to understand the possible effect of education level on the reduction of depression symptoms following SMART. No effects of education level on improvements in anxiety and stress symptoms were found, providing support for SMART as a mental health tool across educational backgrounds.

Generational stress appears to be on the rise. The 2012 Stress in America survey found that Millennials and Gen Xers reported higher average stress levels than those in older generational groups (American Psychological Association, 2012). This trend continued in the 2020 Stress in America survey, finding that Gen Z adults (ages 18–23 in 2020) reported the highest average stress levels of the generational groups (American Psychological Association, 2020). In our study, we found an effect of age on stress that was nearing significance, such that stress may improve more for those of younger ages following SMART, as evidenced by lower symptom endorsement following the online training. This indicates that SMART could be a particularly effective tool for supporting mental health symptoms related to stress for this vulnerable age group. Post hoc testing revealed that younger participants did indeed report more stress symptoms than older participants at baseline (*p* < .001). Although people of all ages experience stress, the sources of that stress and preferred coping mechanisms may differ across the lifespan (Aldwin et al., 1996; American Psychological Association, 2012; Chen et al., 2018). One rising public health concern is the effect of work‐related stress (Leka & Jain, 2010; Ornek & Esin, 2020; Saleem et al., 2021). Because younger participants are more likely to be in the workforce, relative to those of retirement age, it is also possible that SMART could be a particularly effective tool for relieving work‐related stress. Additional exploration of SMART in the workplace could be warranted.

A preliminary exploration of the data from participants who consented to continue in the Project was used to identify if improvements in depression, anxiety, and stress symptoms were maintained 6 months after completion of the training (9 months after baseline assessment). For this subset of participants, findings indicated a significant reduction in depressive and stress symptoms directly following online training access which was maintained 6‐months post‐training. Self‐reported symptoms of anxiety also showed improvement following SMART; however, this effect was not significant until 6 months post training (9 months after baseline assessment). The inability to detect a statistically significant decrease in anxiety symptoms following SMART training for this subgroup could be explained by a ceiling effect in baseline anxiety levels. The participants who completed a pre‐ and post‐training assessment reported a low number of initial baseline anxiety symptoms (*M* = 1.61), and the subgroup who continued on to the longitudinal study reported an even lower level of baseline anxiety symptoms (*M* = 1.39), leaving minimal room for continued improvement. Another explanation for this finding is that there was not enough power to detect a significant effect of change in anxiety directly following the training, as the sample size of participants who completed a 6‐month post‐training follow‐up assessment was reduced to *n* = 44. Similar to results from previous in‐person SMART investigations (Young et al., 2021), overall, these findings do support that mental health benefits are maintained 6‐month post‐training with additional support for application of the strategies via the brain health dashboard.

The effectiveness of online SMART's impact on improving and maintaining mental health symptoms for generally healthy adults provides an opportunity to scale interventions to reinforce brain health at a higher level in the general population. Online SMART allows for reduced interaction time required between participants and providers. In this digital health model, a majority of support offered to the participant is through pre‐generated training materials with limited video conference clinician–coach interactions (about once every 3 months). Therefore, transitioning to offering online mental health supports could increase the number of individuals served by providers and could also reduce the cost of service provision, which is often a barrier for those looking for mental health services (Coombs et al., 2021; Sareen et al., 2007). The online format also increases accessibility, as users do not need to travel to clinicians and can access the self‐paced programing at any time using mobile technology to accommodate varying scheduling needs, such as childcare constraints, working multiple jobs, or working swing shifts.

### 4.1 Limitations

One limitation of this study was the homogeneity of education level within the sample. A majority of participants held a high level of education, that is, having a bachelor's degree or higher level degree. Having a larger number of participants with lower education levels could allow us to better observe education‐based differences in response to SMART. Additionally, the study sample had a high proportion of female participants. Having a more balanced gender sample could allow us to observe other gender‐based training considerations. Another limitation of the current study is the lack of control group. Including a control group of participants who complete the assessment but not the training could provide insight into what effect, if any, can be attributed to the assessment and feedback session versus effects from learning and practicing SMART tactical strategies. A control could have also allowed us to better capture potential improvements attributed to more general life circumstances, such as acclimation to, or improvement in, pandemic‐related conditions impacting their work or home life.

### 4.2 Future directions

Continued exploration is needed to better understand the possible effect of age on stress reduction following online SMART, particularly in elucidating how SMART may lead to resiliency for different types of stressors, such as work‐related stress. Continued investigation with a more educationally diverse sample should also be conducted to examine the effects of education level on the improvement of depression symptoms following SMART. An additional avenue for exploration includes exploring how the utilization of modules and other dashboard resources affect mental health outcomes. As the training was self‐paced, not all participants completed all training modules, whereas others may have even completed modules more than once for additional practice. Future explorations into differing utilization practices may inform our understanding of what “dosage” of SMART could be effective in supporting mental health symptomatology. Another direction is to explore the effects of SMART on mental health for those who have a history of, or are currently experiencing, mental illness to expand our understanding of the scope of individuals who may experience mental health benefits following SMART. Alternatively, it may also be informative to examine the effectiveness of SMART on a targeted study population that has not been diagnosed with mental illness but reports mental illness symptoms. By specifically seeking out a population experiencing mental health symptoms, instead of using a sample from the general population, we may be better able to determine the effect size of the training on mental health symptom changes following SMART. Another important future direction is to examine how changes in cognitive performance may mediate change in mental health symptomatology. This will improve our understanding of the mechanism of change for how SMART, a cognitive intervention, affects mental health symptomatology.

## 5 CONCLUSION

One response to the growing demand for mental health services is to provide the public with resources that create resiliency by increasing mental health before the onset of a true disorder. Online SMART may be an effective tool for the general population, as participants showed a decrease in depression, stress, and anxiety symptoms following the online training. Decreases in self‐reported mental health symptomatology were maintained 6 months post‐training with continued access to SMART, demonstrating the lasting impact of this programing. The online format allows for a decreased clinician workload while still allowing brief personalized coaching sessions. Online training increases accessibility and allows self‐initiated review/continued practice as needed. This novel approach should be considered a low‐cost, high‐impact means to supporting public mental health.

## CONFLICT OF INTEREST

The authors declare that the research was conducted in the absence of any commercial or financial relationships that could be construed as a potential conflict of interest.

### PEER REVIEW

The peer review history for this article is available at https://publons.com/publon/10.1002/brb3.2853.

## Data Availability

The data that support the findings of this study are available from the corresponding author upon reasonable request.
